# The relationship between irritable bowel syndrome and psychiatric disorders: from molecular changes to clinical manifestations

**DOI:** 10.1186/2049-9256-2-4

**Published:** 2014-06-27

**Authors:** Mihaela Fadgyas-Stanculete, Ana-Maria Buga, Aurel Popa-Wagner, Dan L Dumitrascu

**Affiliations:** Department of Neurosciences, Iuliu Hatieganu University of Medicine and Pharmacy, Cluj-Napoca, Romania; Department of Psychiatry, University of Medicine, Rostock, Germany; 2nd Department of Medicine, Iuliu Hatieganu University of Medicine and Pharmacy, Cluj-Napoca, Romania; Department of Molecular Medicine, Iuliu Hatieganu University of Medicine and Pharmacy, Cluj-Napoca, Romania

**Keywords:** Irritable bowel syndrome, Psychiatric disorders, Comorbidities, Brain-gut axis, Psychosocial factors

## Abstract

Irritable bowel syndrome (IBS) is a functional syndrome characterized by chronic abdominal pain accompanied by altered bowel habits. Although generally considered a functional disorder, there is now substantial evidence that IBS is associated with a poor quality of life and significant negative impact on work and social domains. Neuroimaging studies documented changes in the prefrontal cortex, ventro-lateral and posterior parietal cortex and thalami, and implicate alteration of brain circuits involved in attention, emotion and pain modulation. Emerging data reveals the interaction between psychiatric disorders including generalized anxiety disorder, panic disorder, major depressive disorder, bipolar disorder, and schizophrenia and IBS, which suggests that this association should not be ignored when developing strategies for screening and treatment. Psychological, social and genetic factors appear to be important in the development of IBS symptomatology through several mechanisms: alteration of HPA axis modulation, enhanced perception of visceral stimuli or psychological vulnerability. Elucidating the molecular mechanisms of IBS with or without psychiatric comorbidities is crucial for elucidating the pathophysiology and for the identification of new therapeutical targets in IBS.

## Review

### Introduction

Irritable bowel syndrome (IBS) is a functional syndrome characterized by chronic abdominal pain accompanied by altered bowel habits [1, 2]. IBS is a painful condition associated with significant psychological distress and psychiatric comorbidities, like higher levels of anxiety or depression and suicidal ideation, with negative impact on quality of life [2, 3]. Its consequences are measured in direct cost (medical treatment and procedures) and indirect cost (reduced productivity) [4].

The Rome committee published in 1992 the first clinical diagnostic criteria of IBS that could be also used in research. The latest version, Roma III diagnostic criteria, are the current golden standard for diagnosing IBS patients. According to these criteria, IBS is defined as recurrent abdominal pain or discomfort at least 3 days/month in the last 3 months associated with two or more of the following: a) improvement with defecation, b) onset associated with a change in the frequency of stool, c) onset associated with a change in form (appearance) of stool. IBS can be subtyped into categories based on the main bowel habit: IBS with constipation (IBS-C), IBS with diarrhea (IBS-D), mixed IBS (IBS-M), and unsubtyped IBS (IBS-U) [5].

Depending on diagnostic criteria used (Manning, Rome I, Rome II, Rome III), IBS is estimated to affect approximately 11% of the global population. A recent meta-analysis shows the lowest occurrence in South Asia (7%) and the highest in South America (21%) [6]. IBS is reported more frequently by women than men in Western countries, female–male odd ratio being 2:1 and seems to be more common in the ages between 20 and 40. Based on different symptoms observed, the researchers speculated that sex hormones may alter the regulatory mechanisms of the brain-gut-microbiota axis involved in the pathophysiology of IBS [7].

The etiology of this syndrome is multi-factorial and has been attributed to dysregulation of the brain-gut axis, HPA axis, altered gastrointestinal motility, visceral hypersensitivity, infectious factors, enhanced immunological reactivity, genetic susceptibility and psychosocial factors. Communication between central nervous system and enteric nervous system implies a bidirectional connection system: the brain influences the function of the enteric nervous system, and the gut influences the brain via vagal and sympathetic afferents. The symptoms may be caused by dysfunctions either primarily in the central nervous system, or in the gut, or by a combination of both [8–11].

Stress often worsens the symptoms of patients with IBS. Hypothalamic-pituitary-adrenal axis (HPA axis) is an important component of regulatory mechanisms governing behavioural, neuroendocrine and autonomic responses to stress. The hypothalamic-pituitary-adrenal (HPA) axis and the sympathetic nervous system (SNS) are the two major branches of this central stress response system. Hyperactivation of this pathway may be involved in IBS pathophysiology. For example, a hyperreactive responce of the brain and gut to corticotropin-releasing hormone (CRH) and alterations in adrenocorticotropin hormone (ACTH) and cortisol, and catecholamine levels have been reported in IBS patients [12–14]. Indeed, peripheral administration of CRH improved colonic function and visceral perception in response to gut stimulation in IBS patients [13]. In addition, previous studies showed that the IBS is associated with significantly lower vagal tone and increased sympathetic activity [14]. Therefore it has been proposed that the interaction between different factors like brain-gut axis, HPA axis and inflammatory response can exacerbate IBS symptoms and therefore may play an important role in IBS etiology [9].

Gastrointestinal symptoms have been recognized to occur in relation to fear, anxiety and stress. The gut motility has been found to be reactive to changes in the emotional state. Also, stress has been showed to affect both motility and visceral hypersensitivity. Over the past several years, a great deal of research has evaluated the relationship between IBS and psychiatric disorders. Rates of co-morbidity with psychiatric disorders range from 54 to 94% in treatment-seeking patient with IBS [15–17].

Psychiatric disorders and IBS appear to have bidirectional co-morbidities. The frequency and severity of the symptomatology of IBS in patients with anxiety and mood disorders have been well documented. Evolution of IBS symptoms seems to be influenced by affective factors and psychosocial stressors [2, 18]. Those factors may contribute to predisposition, precipitation and maintenance of IBS symptoms, and they also influence the clinical outcome [3, 19]. Psychiatric interventions (pharmacologic or psychotherapy) have proved effectiveness in the improvement of IBS patient functioning. In this review, we emphasize the relationship between IBS and psychiatric disorders.

### Brain imaging studies

Patients with IBS also exhibit differences in brain morphology, when compared with healthy volunteers. IBS patients showed association of disease onset with thicker right posterior insula, but the association with psychological symptoms was not proved [20]. The results of another study support the hypothesis that IBS patients with chronic pain exhibit long-term microstructural changes within the brain, particularly in cortical regions associated with integration of sensorial information and corticothalamic modulation [21]. For example, patients with IBS also showed increased hypothalamic gray matter [22, 23] that may be related to pain-related overstimulation of the hypothalamic-pituitary-adrenal axis. Likewise, IBS was associated with decreased gray matter density (GMD) in different regions of the brain (medial prefrontal cortex, ventro-lateral prefrontal and posterior parietal cortex, ventral striatum, and thalami) [24]. Changes in the density of gray matter among regions involved in cognitive functions are specifically observed in patients with IBS, albeit different levels of anxiety and depression can explain changes in other areas of the brain. The findings of another study on a sample of female IBS patients with moderate symptom severity suggest that morphometric alterations occur primarily in brain networks involved in attention and emotion modulation, as well as in pain modulatory networks, and in systems processing interoceptive information [22].

Changes in gray matter volume and cortical thickness (CT) have been demonstrated in subjects with IBS, especially in female IBS [16, 25]. Also, compared with healthy subjects, in IBS patients, the right part of cingulate gyrus and thalamus are critical for information flow. Brain areas involved in pain modulation were identified as network hubs in IBS [23, 26].

About 15 years ago, functional brain imaging was introduced as a novel method to examine the interactions between gut and brain. The technique has provided the opportunity to assess the neurobiological substrates involved in human behavior and visceral homeostasis. Recent studies in neuroscience have shown that the brain functions involved in emotion are modulated by visceral pain. The researchers identified two important neural networks with abnormal functioning: the lateral prefrontal regions of the descending pain inhibition network, and the emotion-arousal networks. Furthermore functional imaging studies may provide relevant information about cognitive mechanisms, which may be involved in neurobiological abnormalities of IBS [27, 28].

Functional MRI (fMRI) studies point to dysfunction of emotion and attention processing of pain in IBS. Patients with IBS pain can be verbally programmed to produce placebo analgesia by decreased brain activity related to cerebral mechanisms involved in memory and semantic processing [29]. Since then, an increasing number of imaging studies assessed the brain activity in regions involved in processing the visceral homeostatic afferent input and have shown abnormal activity in different regions of the brain (anterior/mid cingulate cortex primary and secondary somatosensory cortex, insular cortex, thalamic and prefrontal cortex).

The affective component of physical pain is processed cortically by the dorsal anterior cingulate cortex and the anterior insula. Both regions have some of the highest numbers of mu-opioid receptors in the central nervous system [30]. The perception of pain is modulated by several factors like affective and sensory aspects of the painful stimulus, coping strategies and personality features [31, 32]. Catastrophic thinking increases the frequency of maladaptive thoughts about pain and neuroticism is correlated with increased sensitivity to negative cues.

A quantitative meta-analysis of available data revealed that patients with IBS appears to show greater activation of amygdale and perigenual anterior cingulate gyrus nodes, but similar activation of regions involved in the processing of visceral afferent information, as compared with healthy controls [32]. Further, patients with IBS have increased activity in the insula and decreased activation of the dorsolateral prefrontal cortex in response to visceral stimulation [33, 34].

One recent study investigated if the patients have impairments in cognitive flexibility due to dysfunction in the dorsolateral prefrontal cortex and insula, and altered connectivity between brain regions. Event-related fMRI of the brain was performed to evaluate cognitive flexibility and was assessed by the Wisconsin Card Sorting Test (WCST). The subjects with IBS presented significantly more perseverative errors, and set-maintenance difficulties than controls. The results showed significantly increased activity of the left posterior insula at error feedback during set-shifting, and significantly decreased activity of the right dorsolateral prefrontal cortex, and right hippocampus in subjects with IBS [34].

Some other studies showed that in IBS, rectal distention determine anterior cingulate cortex (ACC) activation that correlates with anxiety, maladaptive coping, stressful events and a history of abuse. IBS diagnosis and abuse history appear to have synergistic effects causing even greater activation of the perigenual pACC. These studies support clinical observations regarding the connections between psychological distress, IBS, and increased pain [35].

### Panic disorder and IBS

Panic disorder (PD) is a frequent psychiatric disorder, with a lifetime prevalence between 1.5% and 3.5%. Agoraphobia is present in 20,2% of patient affected by PD [36, 37].

High comorbidity between IBS and PD has been identified in the literature [3, 17, 38]. The prevalence of IBS symptoms characteristics in patients with PD varies between 25 to 44%, several symptoms being characteristic of the two disorders (nausea, diarrhea, abdominal discomfort) [39–42].

Recent studies tried to clarify the nature of the relationship between PD and IBS. One explanatory model is represented by the functional relationship between the central nervous system (CNS) and enteric nervous system (ENS). Much of the results of recent research suggest that the dysregulation of hypothalamic-pituitary-adrenal axis (disruption of the normal inhibitory feed-back) may represent a common pathway that may lead to stress vulnerability [41, 43, 44]. Patients with IBS and anxiety disorders present abnormally elevated basal cortisol levels and altered immune function (increased cytokines levels) [45–47].

There is evidence that the presence of avoidant behavior because of fear of IBS symptoms may be linked to a more severe form of agoraphobia, and the latter may also be associated with depression. The results of a recent study imply that presence of IBS may be related to anticipatory anxiety and agoraphobia in patients with PD [47, 48].

### Generalised anxiety disorder and IBS

Gastrointestinal-specific anxiety seems to perpetuate IBS symptoms through disruption of autonomic and pain facilitation, as well as cognitive mechanisms. Anticipatory worries and avoidance behavior are the main reason of affected functioning [49, 50]. One study examined the relationship between IBS and GAD among IBS patients and healthy subjects and 32% of subjects with IBS presented GAD symptoms as compared with other psychological manifestations [50]. According to this study, visceral sensitivity score (VSI) is the single powerful predictor of the severity of gastrointestinal-specific anxiety (GSA). Another study examined the prevalence, comorbidity and risks correlates of IBS in a general population and confirmed that there is a strong association between IBS and GAD, i.e., patients with comorbid IBS-GAD had more functional impairment and had more depressive symptoms [51].

Both diseases may benefit from treatment with antidepressants (selective serotonin reuptake inhibitors-SSRIs). Serotonin stimulates gastro-intestinal motility and visceral perception at CNS level by activating the serotonin receptor. SSRIs used for anxiety disorders and/or depression can improve the outcome of patients with IBS and associated psychiatric disorders, in special with constipation-predominant IBS form. However, is not entirely clear if the mechanism underlying IBS are linked to the drugs effects on mood [52].

Also, the results of a recent pilot study support the efficiency of duloxetine treatment in comorbid IBS and GAD. Patients reported significant reductions in IBS symptoms, as well as improvement in GAD [53].

### Post traumatic stress disorder and IBS

A number of studies have consistently reported a relationship between history of various type of abuse and IBS. It is not clarified yet what is the connection between posttraumatic stress disorder (PTSD) and IBS. Much of the research had been focused on the role of sexual abuse as a risk factor to acquire IBS with most studies reporting an increased IBS risk [54, 55].

Another study reported that 36% of patients with IBS have met lifetime diagnostic criteria of PTSD [56]. Other authors reported a substantially increased risk for IBS among female veterans diagnosed with PTSD or for military after deployment to war [57, 58]. Indeed, one case study demonstrated that treating PTSD symptoms first may contribute to an improvement in IBS symptoms [59].

### Major depressive disorder and IBS

One of the most diagnosed psychiatric disturbances in IBS patients is depression. There are many studies that evaluated the prevalence of anxiety and depression among patients with IBS seeking care in gastroenterology units but few studies have been done on IBS in psychiatric patients. An increased prevalence (27–47, 3%) of IBS in patients with major depression (onset or recurrent episode) was reported by several studies [60, 61]. More recently, a cross-sectional study investigated the prevalence of IBS symptoms in patients diagnosed with major depressive disorder (MDD) [62]. The results demonstrated a higher prevalence of IBS symptoms in patients with depression compared with healthy subjects but patients with MDD in remission did not differ from healthy controls in reporting gastrointestinal symptoms.

Females with IBS have abnormal increased tryptophan degradation along the kynurenine pathway due to upregulation by proinflammatory cytokines. The rapid degradation of tryptophan depletes tryptophan and serotonin and produces toxic metabolites. This mechanisms could represent a possible biological basis for the high co-morbidity between IBS and depressive and anxiety disorders [63, 64].

### Bipolar disorders and IBS

There are very few studies assessing the association between bipolar disorder and IBS. One community study found a lack of association between bipolar disorder and IBS [65]. Another case study reported no significant relationship between mood and IBS symptom severity [66].

### Schizophrenia and IBS

Schizophrenia is a chronic disease associated with a high-risk of developing comorbid somatic illnesses. Somatic co-morbidities represent a major issue in patients with schizophrenia. The prevalence of IBS in schizophrenia patients was reported by Gupta and colleagues, to be 17% [67]. Another study reported a prevalence of 19% [68]. One of the main findings of this study was that patients with schizophrenia rarely complain about IBS symptoms, unless specifically asked. In patients with schizophrenia there may be significant barriers in obtaining adequate health care when comorbid disorders occur. These obstacles may be the result of patient psychotic symptomatology, stigma, and financial problems. It should be remembered that, under these circumstances, the actual prevalence of IBS symptoms may be higher, as these results are dependent upon individual reporting of symptoms.

### Alcohol abuse/dependence and IBS

The findings relating alcohol abuse and IBS are contradictory. In population-based studies, alcohol abuse was not found to be associated with IBS. Moreover, the majority of patients with IBS tried to avoid certain food in order to reduce their symptoms, including alcohol [69]. Still another study conducted in a psychiatric setting concluded that IBS is underdiagnosed in patients with alcohol abuse and alcohol dependence [61]. The findings of a recent study investigating the role of alcohol ingestion on gastrointestinal symptoms provided indication that it is the pattern of alcohol consumption may influence symptomatology in IBS patients [70].

### Genes involved in ibs and psychiatric comorbidities

The pathogenesis of IBS associated with psychiatric comorbidities appears to have multifactorial aspects. Several factors appear to play a role in this process, such as psychological factors, genetic factors, chronic intestinal inflammation, and/or altered signaling in CNS and gut neuroendocrine system (NES) (Table 1). The IBS is not a “single gene” disease, many factors involved in IBS (e.g. individual vulnerability, psychosocial stressors) can be modulated by targeting these diseases at molecular level (interaction between genes and/or signaling pathway modulation in response to environment).

**Table 1:** **Potential target genes associated with psychiatric diseases in IBS**

Gene symbol	Description	Gene function	Gene expression in IBS	Psychiatric diseases	Reference
NGF	Nerve growth factor	Pro-inflammatory mediator; psychoneuroendocrine modulator	Overexpression (in rat model)	Anxiety, chronic alcohol consumption, depression	[71, 72]
BDNF	Brain-Derived Neurotrophic Factor	Regulation of stress response and in the biology of mood disorders	SNP	Psychiatric IBS, schizophrenia, mood disorders, PTSD	[73, 74]
COMT	Catechol	Degradative pathways of the catecholamine transmitters; metabolism of catechol drugs	SNP	Anxiety, panic disorder, and cognitive performance	[75, 76]
	O-Methyltransferase				
OPRM1	Mu Opiate Receptor	Principal target of endogenous opioid peptides	SNP	Pain sensitivity, opioid dependence, and social sensitivity	[77, 78]
5-HTTLPR	5-hydroxytryptamine transporter gene-linked polymorphic region	Serotonin transporter	SNP	Depression, anxiety, rectal pain ratings	[79, 80]

Neurotrophins like NGF (nerve growth factor) or Brain-derived neurotrophic factor (BDNF) are involved, not only in somatic and visceral hypersensitivity, but also in neuronal survival, maturation and migration in the peripheral and central nervous system (CNS). Previous studies have reported a massive release of NGF in the peripheral circulation together with increased mRNA level in hypothalamus to integrate behavioral and neuroendocrine response following a psychosocial stress. Systemic administration of an anti-NGF antibody can prevent colonic hypersensibility and can be considered as a potential new therapeutic target [71, 72].

BDNF promote neural plasticity and plays an important role in the modulation of stress response and pathophysiology of mood disorders. Many studies were focused on the genetic polymorphisms associated with IBS. Yu and colleagues showed that the increased BDNF expression as compared with healthy controls in gut biopsies is associated with IBS [73]. On the other hand, BDNF Val66Met single nucleotide polymorphism (BDNF Val66Met SNP) was associated with decreased BDNF secretion from neurons and may lead to impairments in learning and altered susceptibility to stress [74].

Polymorphisms that affect many systems like serotonergic, adrenergic and opioidergic systems may also be involved in IBS pathophysiology. Adrenergic and opioidergic systems are involved in the modulation of motor and sensory function of gut and their polymorphism has been correlated with differential response to pain and with variation in alteration of gut motility [75–78].

Serotonin transporter gene (SERT) polymorphisms are associated with higher risk of depression in IBS patients. Thus individuals with a diminished function of serotonin transporters are more sensitive to gut signals in emotion-regulating brain regions [79, 80].

## Conclusions

A number of psychiatric comorbidities affect the patients with IBS. In particular, anxiety disorders and mood disorders occur with a significant greater frequency, indicating that the assessment or treatment of these comorbid conditions may influence the outcome of IBS. Although symptoms may improve with pharmacologic treatment, the residual manifestations of IBS may still impact the social and occupational functioning. Elucidating the molecular mechanisms of IBS with or without psychiatric comorbidities is crucial for elucidating the pathophysiology and for the identification of new therapheutical targets in IBS. A lot of data suggest the benefit of adjunctive psychological and genetical interventions but further investigation of such interventions is required.
